# Synthesis and Characterization of Ternary α-Fe_2_O_3_/NiO/rGO Composite for High-Performance Supercapacitors

**DOI:** 10.1021/acsomega.2c02418

**Published:** 2022-08-01

**Authors:** Geerthana Mummoorthi, Shanavas Shajahan, Mohammad Abu Haija, Umadevi Mahalingam, Ramesh Rajendran

**Affiliations:** † Department of Physics, Periyar University, 636 011 Salem, Tamil Nadu, India; ‡ Department of Chemistry, Khalifa University, P.O. Box, 127788 Abu Dhabi, United Arab Emirates; § Center for Catalysis and Separations, Khalifa University of Science and Technology, P.O. Box., 127788 Abu Dhabi, United Arab Emirates; ∥ Department of Physics, Mother Teresa Women’s University, 624 10 Kodaikanal, Tamil Nadu, India

## Abstract

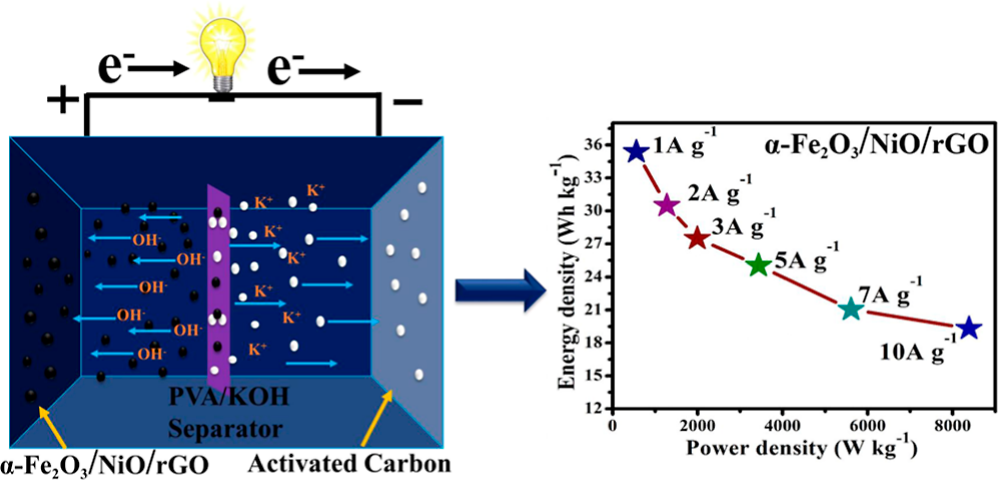

Herein, pure α-Fe_2_O_3_, binary α-Fe_2_O_3_/NiO, and ternary α-Fe_2_O_3_/NiO/rGO composites were prepared by a hydrothermal method. The properties of the prepared materials were studied by powder X-ray diffraction, scanning electron microscopy, TEM, XPS, and Brunauer–Emmett–Teller techniques. The clusters of smaller α-Fe_2_O_3_ nanoparticles (∼30 nm) along with conducting NiO was freely covered by the rGO layer sheet, which offer a higher electrode–electrolyte interface for improved electrochemical performance. The ternary composite has shown a higher specific capacitance of 747 F g^–1^@ a current density of 1 A g^–1^ in a 6 M KOH solution, when compared with that of α-Fe_2_O_3_/rGO (610 F g^–1^@1 A g^–1^) and α-Fe_2_O_3_ (440 F g^–1^@1 A g^–1^) and the nanocomposite. Moreover, the ternary α-Fe_2_O_3_/NiO/rGO composite exhibited a 98% rate capability @ 10 A g^–1^. The exceptional electrochemical performance of ternary composites has been recognized as a result of their well-designed unique architecture, which provides a large surface area and synergistic effects among all three constituents. The asymmetric supercapacitor (ASC) device was assembled using the ternary α-Fe_2_O_3_/NiO/rGO composite as the anode electrode (positive) material and activated carbon as the cathode (negative) material. The ASC device has an energy density of 35.38 W h kg^–1^ at a power density of 558.6 W kg^–1^ and retains a 94.52% capacitance after 5000 cycles at a 1 A g^–1^ current density.

## 1 Introduction

Electronic-based digital communications, electric cars (bus), burst power production applications, and memory backup-based devices that require high-power pulses in a short length of time are all examples of electrochemical purposes.^1,2^ In recent years, the energy crisis has sparked a lot of interest in electrochemical supercapacitors due to the anticipated demand driven by energy conversion and integrated energy storage systems.^3^ Under these circumstances, electrochemical energy storage systems are consolidated along with renewable energy sources to store energies and also deliver efficiently.^4^ Exploring nanotechnology for the next generation has revealed progress in higher performance electrochemical devices, particularly electrochemical supercapacitors emerging from energy conversion and storage systems.^5^ The supercapacitors are known for their quick charging and discharging capabilities. It also has a high power density along with long cycle life. Electrochemical energy storage systems are preferred due to their low cost and ease of maintenance.^6^ Supercapacitors are divided into two types depending on the charging storage mechanisms as well as electrode materials: (i) electrochemical double-layer capacitors (EDLCs) and (ii) pseudocapacitors.^7^ Transition metal oxides with high electrical resistance are often employed as pseudocapacitor electrode materials because of their improved reduction and oxidation reversibility and higher theoretical specific capacitance.^8^ For example, RuO_2_,^9^ α-Fe_2_O_3_,^10^ NiO,^11^ Co_3_O_4_,^12^ WO_3_,^13^ MnO_2_,^14^ ZnO,^15^ and CuO,^16^ and so forth, have been investigated as pseudocapacitor electrode materials. Recently, α-Fe_2_O_3_ has been explored as a promising candidate for positive electrode materials due to its variable oxidation states, good stability, and thermodynamically stable structure.^4,17^ In practical, α-Fe_2_O_3_ has lower ionic and electronic transport properties prone to decrease the cyclic stability and suppress the electrochemical reaction kinetics at a large current density.^18^

The doping or composite with nanomaterials can increase the surface area to increase the electrochemical reaction sites. Graphene exhibits an ultrathin structure, a high specific surface area, an ideal mechanical strength, and an excellent electrical conductivity.^19^ According to Chen et al., the graphene sheet prevents the agglomeration of α-Fe_2_O_3_ nanoparticles during the synthesis, resulting in high cycle stability and rate capability.^20^ The decoration of iron oxide on nitrogen-doped graphene improved the specific capacitance of α-Fe_2_O_3_ nanoparticles in an alkaline electrolyte solution exploring with superior capacitance retention (about 75.3% at 5 A g^–1^) even after 100,000 cycles. Liu et al.^7^ improved the electrochemical stability of the α-Fe_2_O_3_ electrode material by incorporating NiO with improved ion/electronic transport properties.^21^ Jiao et al. reported the synthesis of hybrid α-Fe_2_O_3_@NiO heterostructures on a carbon cloth by a hydrothermal method and demonstrated the enhanced specific capacitance.^22^ Therefore, the formation of the α-Fe_2_O_3_ composite could be expected to improve the overall electrochemical properties of α-Fe_2_O_3_.^23^ Sahoo and Shim fabricated a ZnCo_2_O_4_/r-GO/NiO composite film on nickel foam and demonstrated that the three-dimensional ternary composite showed a specific capacitance of about 1256 F g^–1^ at 3 A g^–1^.^24^ Moyseowicz et al. studied the electrochemical performance of the polypyrrole/Fe_2_O_3_/r-GO composite, which showed a high specific capacitance of 140 F g^–1^.^25^ Based on the aforementioned discussion, the electrochemical properties of the earth-abundant metal oxides could be improved by formation of composites with other metal oxides and conduction carbon scaffold.^26^

Herein, the ternary α-Fe_2_O_3_/NiO/rGO composite was synthesized through a hydrothermal method and a hybrid microwave annealing process. The hybrid microwave annealing furnace has been equipped with controlled on–off cycles of the magnetron to create a microwave field, and temperature was measured using IR sensors.^27−32^ Further, the properties of the prepared materials were studied by powder X-ray diffraction (PXRD), scanning electron microscopy (SEM), TEM, XPS, and Brunauer–Emmett–Teller (BET)-surface area measurements. Moreover, the electrochemical properties of the prepared materials were studied in three electrode configurations. Finally, we assembled an asymmetric supercapacitor (ASC) using synthesized materials as the anode and activated carbon (AC) as the cathode, and then the device performance was studied.

## 2 Materials and Methods

### 2.1 Materials

Pristine graphite powder (≤20 μm, 99%), ferric nitrate nonahydrate (Fe(NO_3_)_3_·9H_2_O), hydrogen peroxide (H_2_O_2_), potassium permanganate (KMnO_4_), and sodium nitrate (NaNO_3_) were purchased from Sigma-Aldrich. Nickel nitrate hexahydrate (Ni(NO_3_)_2_·6H_2_O), sodium dodecyl sulfate (SDS-C_12_H_25_O_4_SNA)-SDFCL, and urea (CH_4_N_2_O) were purchased from Loba Ltd. Ethanol (CH_2_CH_2_OH, 99%) and sulfuric acid (H_2_SO_4_, 98%) were purchased from Rankem, India.

### 2.2 Preparation of Pure α-Fe_2_O_3_

**Figure 1:**
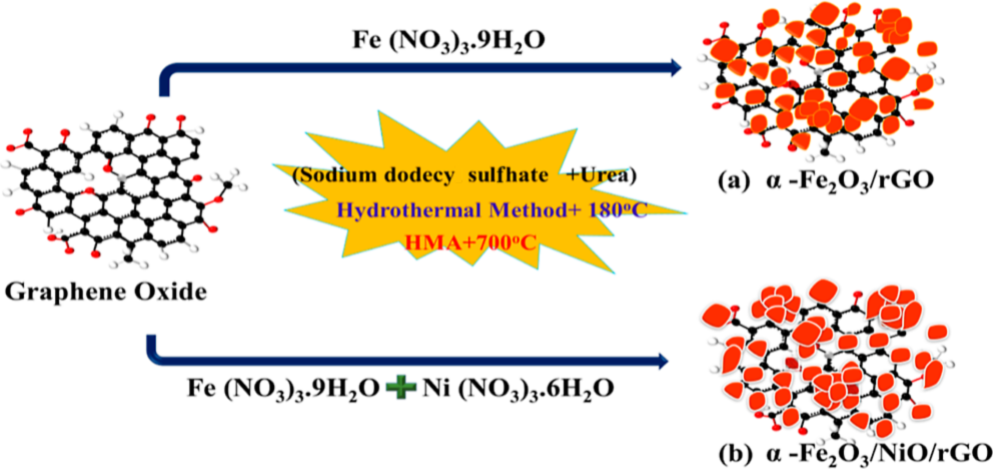
Schematic illustration for the preparation of (a) binary α-Fe_2_O_3_/rGO and (b) ternary α-Fe_2_O_3_/NiO/rGO composites.

The overall synthesis method is explained in the schematic diagram shown in Figure 1. In Brief, the precursor solution was prepared by dissolving sodium dodecyl sulfate (1 g), urea (0.8 g), and ferric nitrate (2 g) mixed in 80 mL of deionized water and allowing for 1 h of stirring at room temperature. The blended slurry solution was then placed into a Teflon-lined stainless steel autoclave and heated at 180 °C for 5 hours for hydrothermal reaction. To remove the unreacted material, the finished product was centrifuged with water and ethanol before being dried in a hot-air oven for 12 h at 80 °C. The final product was subsequently decomposed under a hybrid microwave furnace (700 °C −10 min) to form a black α-Fe_2_O_3_ powder upon annealing.

### 2.3 Preparation of Binary α-Fe_2_O_3_/rGO and Ternary α-Fe_2_O_3_/NiO/rGO Composites

Binary and ternary composites were prepared via the hydrothermal method. Graphene oxide (GO) was synthesized by modified Hummer’s method.^28,29^ In the binary α-Fe_2_O_3_/rGO composite synthesis, an appropriate amount sodium dodecyl sulfate (1 g), urea (0.8 g), and ferric nitrate (2 g) in 40 mL of deionized water were added to GO suspension under continuous stirring. In the typical synthesis of the ternary α-Fe_2_O_3_/NiO/rGO composite, 2 g of nickel nitrate was added with the solution which we used in the synthesis of binary α-Fe_2_O_3_/rGO composite. Then, the solution was then transferred to a 100 mL Teflon-lined stainless steel autoclave and autogenously pressured for 5 h at 180 °C. After the hydrothermal reaction, the obtained binary and ternary product was collected by centrifugation and dried in a hot air oven. Then, the dried product was calcined in a hybrid microwave furnace (HMA) at 700 °C for 10 min to obtain α-Fe_2_O_3_/rGO and ternary α-Fe_2_O_3_/NiO/rGO composites.

## 3 Materials Characterization

The crystalline phase of the obtained samples was analyzed by X-ray diffraction (Smart Lab, Rigaku Corporation Ltd.) using the Cu Kα as X-ray source. The morphology of the synthesized materials was studied using field-emission SEM (FE-SEM) (Supra 40, Carl Zeiss) with an accelerating voltage of 15k attached with energy-dispersive spectroscopy (EDS). The internal morphology was analyzed through HRTEM (JEOL, JEM-2100F), performance at 200 kV). The chemical states and binding energy were studied using the X-ray photoelectron spectroscopy (XPS) technique (ESCA 3400 spectrometer). BET was used to measure and compute the specific surface area and pore size distribution under nitrogen adsorption–desorption isotherms (BET, Micromeritics, ASAP—2020, USA).

## 4 Electrochemical Studies

The electrochemical characterization of three-electrode systems was assessed using an electrochemical workstation (SP-150, Bio Logic Science). All of the experiments were carried out with an aqueous KOH 6 M alkaline electrolyte solution. Cyclic voltammetry (CV) was set in an operational potential window range of 0.0–0.4 V (vs Ag/AgCl) at varied scan rates. Chronopotentiometry behavior was used to create GCD curves from 1 to 10 A g^–1^. Electrochemical impedance spectroscopy (EIS) was performed in the frequency range of 1 Hz to 1 MHz using a 5 mV applied amplitude at an open-circuit potential.

The positive electrode was fabricated by coating the slurry pre-cleaned pressed nickel foam (NF) containing active material (80%), acetylene black (10%), polyvinylidene fluoride (10%), and *N*-methyl-2-pyrrolidone organic solvent. Then, the coated electrode was dried for 7 h at 60 °C in a hot air oven. The mass loading of active materials was calculated to be ∼1 mg. The electrochemical measurements were performed in a three-electrode configuration, where the fabricated electrode was used as the working electrode, Ag/AgCl was used as the reference electrode, and platinum wire was used as the counter electrode.

The specific capacitance of the prepared electrodes was calculated from the GCD curve by eq 1.^30^
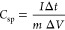
1where *C*_sp_ is the specific capacitance (F g^–1^), *I* is the applied current (A), Δ*t* is the discharge time (s), *m* is the active mass in the electrode (g), and Δ*V* is the working potential window measured (V).

The ASC device was assembled using the ternary α-Fe_2_O_3_/NiO/rGO composite as the positive electrode and AC as the negative electrode materials. The electrodes were prepared by gentle coating of the ternary α-Fe_2_O_3_/NiO/rGO composite or AC on a 1 cm radius disc-shaped NF as described in the previous section. Further, the PVA/3.0 M KOH gel electrolyte was prepared by mixing 3.0 M KOH in PVA polymeric solution. The ASC device was assembled in a Swagelok-type cell using the fabricated anode, cathode, gel electrolyte, and spacer (Whatman filter paper). The electrochemical properties of the prepared supercapattery were studied by CV, GCD, and EIS techniques.

The specific capacitance of the ASC device was calculated using the following equation
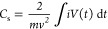
2

The energy density (*E*) and power density (*P*) of ASC was determined from the GCD curve and computed using eqs 3 and 4, respectively
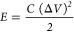
3
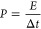
4where *C*_s_ (F/g) is the specific capacitance, *I* (A) is the discharge current, Δ*V* (V) denotes the potential window, Δ*t* (s) is the discharge time measured in seconds, and *m* is the total active mass (g) of ASC (positive and negative) materials.^31^

## 5 Results and Discussion

Figure 1 shows the schematic representation of the synthesis of binary α-Fe_2_O_3_/rGO and ternary α-Fe_2_O_3_/NiO/rGO composites. During the hydrothermal reaction, auxiliary reagents (urea and SDS) support the formation of positively charged Fe- and Ni-based structures^32^ and then attached with the negatively charged GO sheet’s surface to form the metal oxides (α-Fe_2_O_3_ and NiO).^33^ Finally, the obtained materials were heated by the HMA process at 700 °C for 10 min.

**Figure 2:**
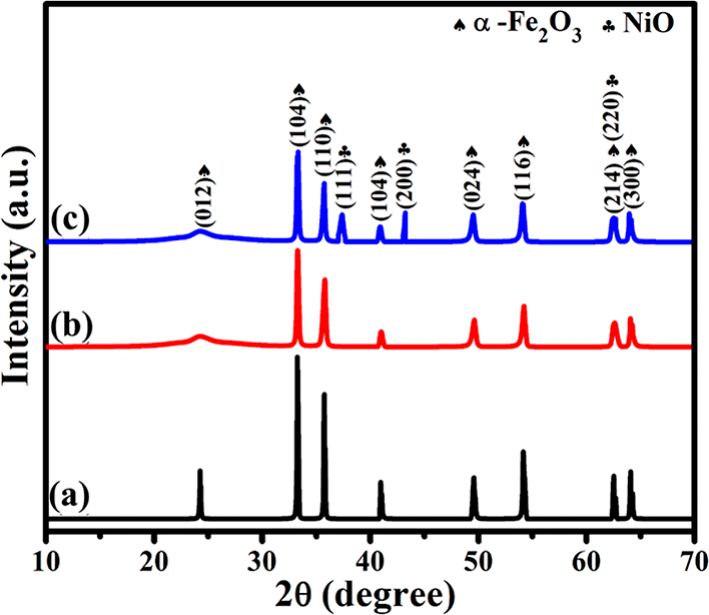
XRD patterns of (a) pure α-Fe_2_O_3_, (b) binary α-Fe_2_O_3_/rGO, and (c) ternary α-Fe_2_O_3_/NiO/rGO composites.

The crystal phase and structure of hydrothermally synthesized pure (α-Fe_2_O_3_), binary (α-Fe_2_O_3_/rGO), and ternary (α-Fe_2_O_3_/NiO/rGO) composites were determined by the PXRD pattern, as shown in Figure 2. Figure 2a shows that the diffraction peak of α-Fe_2_O_3_ at 24.13, 33.15, 35.61, 40.85, 49.48, 54.09, 62.45, and 63.99° corresponds to the (0 1 2), (1 0 4), (1 1 0), (113), (0 24), (1 1 6), (21 4), and (3 0 0) crystal planes, respectively. The XRD patterns of α-Fe_2_O_3_ were shown in the rhombohedral crystal phase, which is well consistent with JCPDS card no. 33-0664. In the ternary α-Fe_2_O_3_/NiO/rGO composite, the planes (111) and (200) at 37.24 and 43.27° were obtained along with characteristic peaks of α-Fe_2_O_3_, which were related to the face-centered cubic structure of NiO and were well consistent with JCPDS card no. 47-1049. The characteristic peak of α-Fe_2_O_3_ at 24.13° was slightly shifted and broadened in the XRD patterns of binary α-Fe_2_O_3_/rGO and ternary α-Fe_2_O_3_/NiO/rGO composites. This result may be due to the inclusion of rGO in the composites (Figure 2b,c). However, the peak related to rGO did not appear in the XRD patterns of the composite due to the high dispersion of α-Fe_2_O_3_ and NiO nanoparticles on the rGO sheet and low weight percentage.

**Figure 3:**
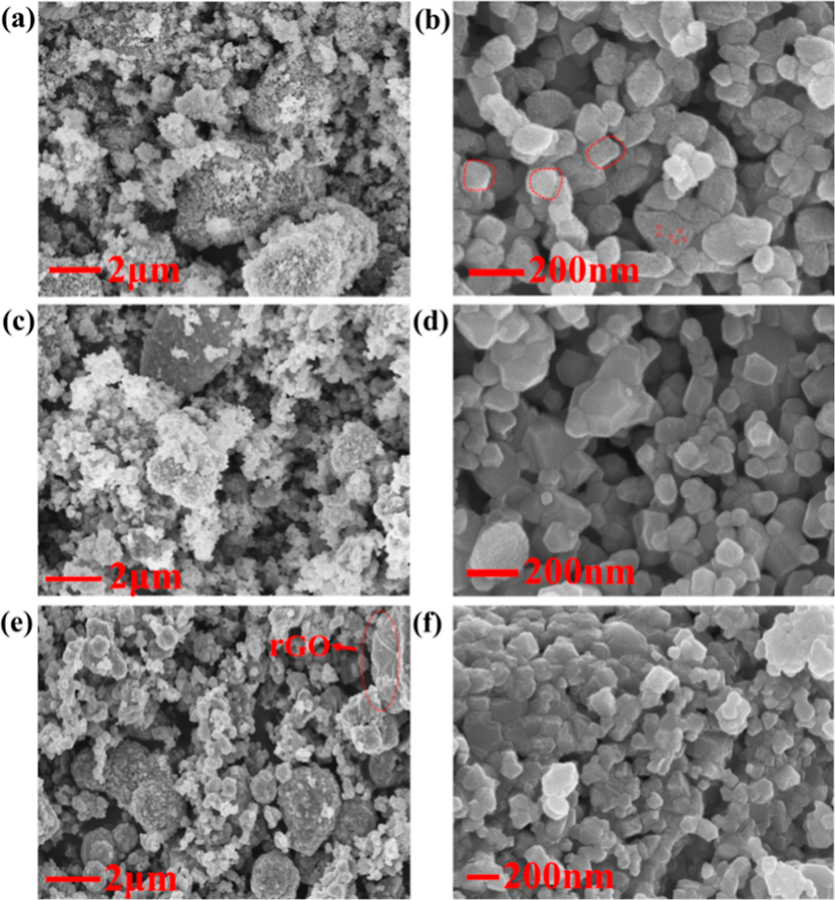
FE-SEM images of (a,b) pure α-Fe_2_O_3_, (c,d) binary α-Fe_2_O_3_/rGO, and (e,f) ternary α-Fe_2_O_3_/NiO/rGO composites.

Figure 3a,b shows the SEM image of an irregular cube-like α-Fe_2_O_3_ structure that is made up of small nanoparticles with size around 10 nm and formed a mesoporous structure (Figure 1). The approximate size of the cube-like α-Fe_2_O_3_ was measured as ∼80 nm. The porosity of the cube-like α-Fe_2_O_3_ structure drastically diapered as a result of surface attachment of rGO (Figure 3b,c).^34^ The density of the particles was increased in the case of ternary α-Fe_2_O_3_/NiO/rGO composites (Figure 3e,f).

**Figure 4:**
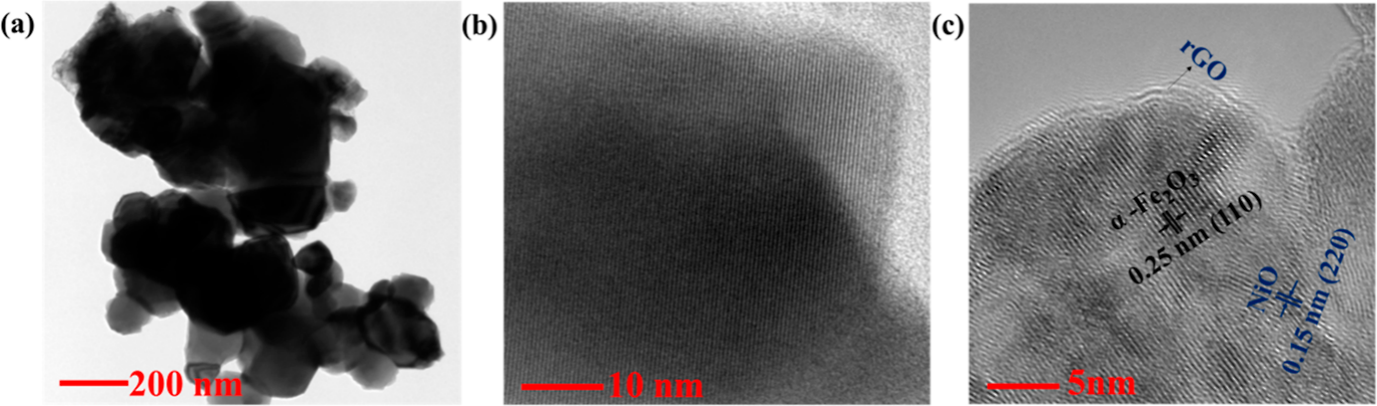
(a) TEM image and (b,c) HRTEM (lattice fringes) of the synthesized ternary α-Fe_2_O_3_/NiO/rGO composite sample.

The internal morphology and crystal structure of the prepared materials were identified by TEM and HRTEM, as shown in Figure 4a–c. In the TEM image of the ternary α-Fe_2_O_3_/NiO/rGO composite, two different particles could be seen; one is in an irregular cube-like morphology which is dark in nature with a broader size (α-Fe_2_O_3_) and the other which is bright in nature with a smaller size (NiO). In addition, the rGO sheet was wrapped on the surface of both materials to form the ternary α-Fe_2_O_3_/NiO/rGO composite. The distinct lattice planes could be seen in the HRTEM image with two different orientations and spacings measured as 0.25 and 0.15 nm, which corresponds the (110) and (220) planes for the rhombohedral crystal phase of α-Fe_2_O_3_ and the cubic crystal phase of NiO, respectively.

**Figure 5:**
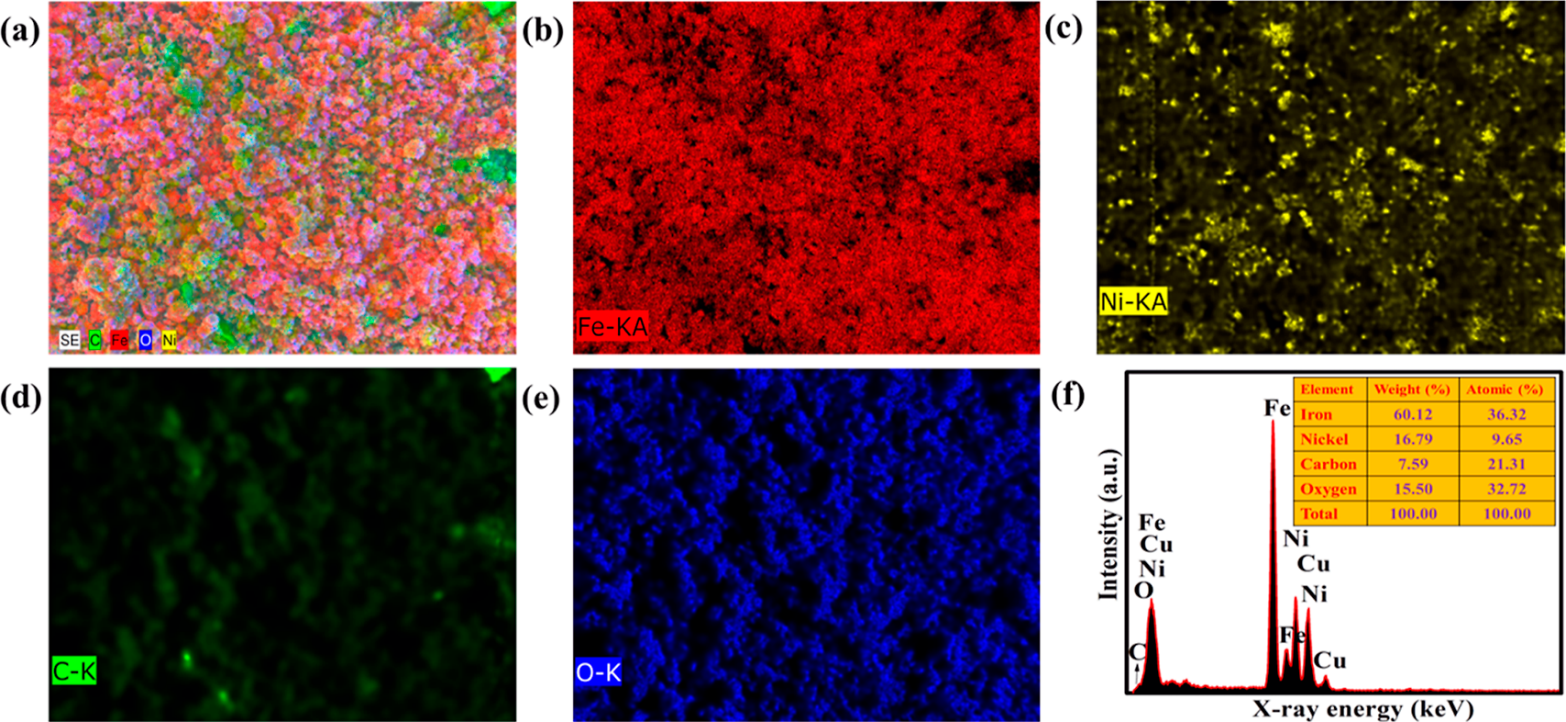
Elemental mapping of (a) survey, (b) Fe atoms, (c) Ni atoms, (d) C atoms, (e) O atoms, and (f) EDS spectrum of the ternary α-Fe_2_O_3_/NiO/rGO composite.

EDS mapping confirms the existence of iron (Fe), nickel (Ni), carbon (C), oxygen (O), and copper (Cu) in the ternary composite sample (Figure 5a–e). The EDS signal from Cu was originated from the copper grid used in the TEM analysis.

**Figure 6:**
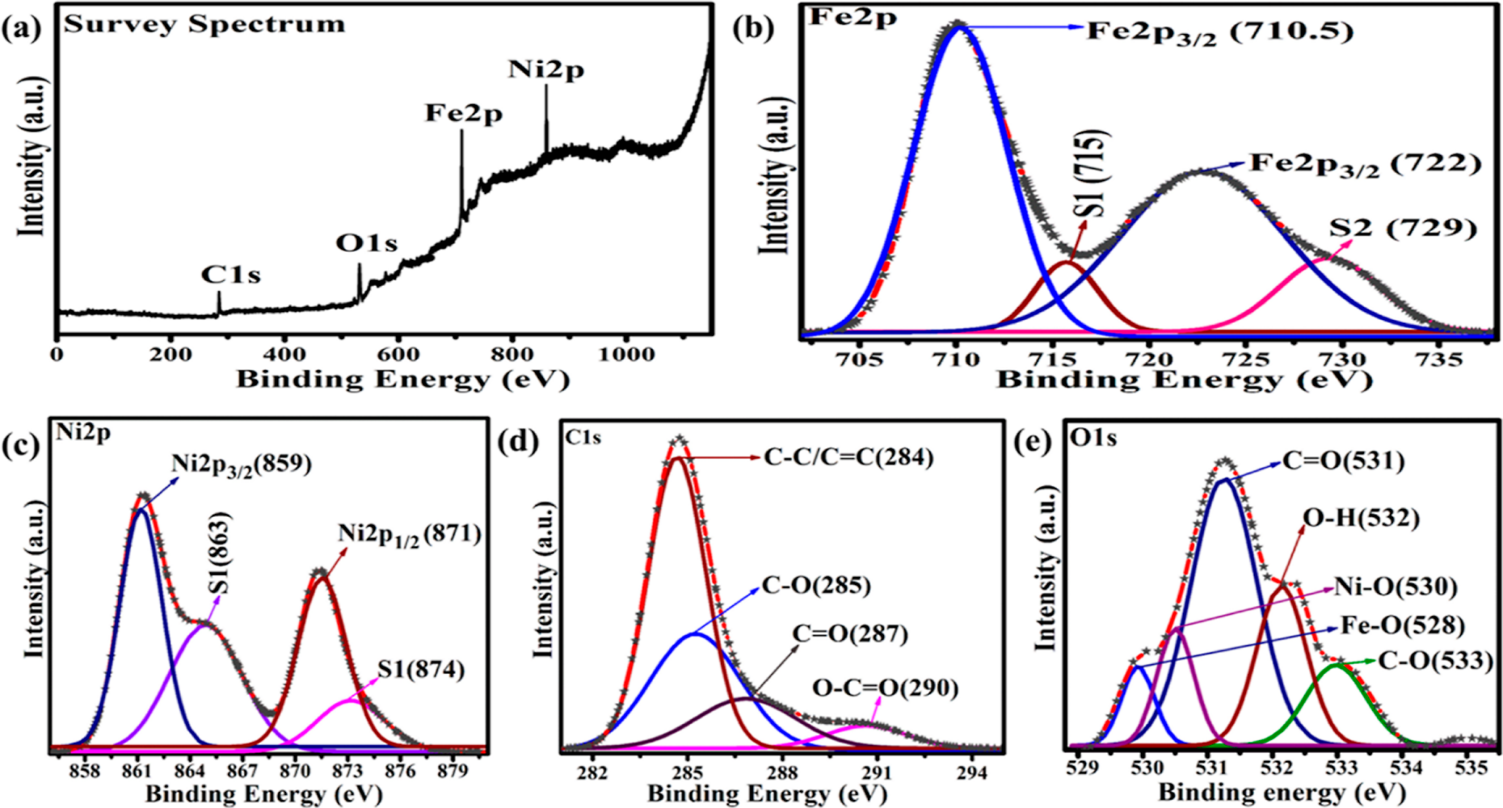
XPS (a) survey, (b) Fe 2p, (c) Ni 2p, (d) C 1s, and (e) O 1s spectra of the ternary α-Fe_2_O_3_/NiO/rGO composite.

The XPS spectrum was used to examine the chemical sates and composition of the ternary α-Fe_2_O_3_/NiO/rGO composite. Figure 6a shows the XPS survey spectrum of ternary α-Fe_2_O_3_/NiO/rGO composite, and the presence of Fe, Ni, C, and O has been observed. The core-level spectrum of Fe 2p was split into two peaks and was assigned to Fe 2p_3/2_ and Fe 2p_1/2_ chemical states. Moreover, the two shakeup satellite peaks centered at 710 eV (S1) and 729 eV (S2), respectively (Figure 6b). Further, the Ni 2p spectrum was deconvoluted into Ni 2p_3/2_ (∼859 eV) and Ni 2p_1/2_ (∼871 eV) with the satellite peaks at binding energies of 863 eV (S1) and 874 eV (S2).·As shown in Figure 6d, the core-level C 1s spectrum consists of five separate peaks at 284 eV for C=C/C–C, 287 eV for C–O, 288 eV for C–O, and 290 eV for C=O and O–C=O groups. The phenolic functional groups in reduced graphene oxide were discovered by deconvolution of the singlet O 1s peaks in Figure 6e. The highly intense peak at 528 eV (Fe–O) and 530 eV (Ni–O) links to the oxygen species (O 1s) in the metal oxide lattice. Peaks at 533 eV for C–O (oxygen single bond to carbon), 531 and 532 eV for C=O (oxygen double bond to aromatic carbon) linkage peaks revealed OH^–^ radical and absorbed H_2_O molecules.^35^ These results confirm the formation of the ternary α-Fe_2_O_3_/NiO/rGO composite.

**Figure 7:**
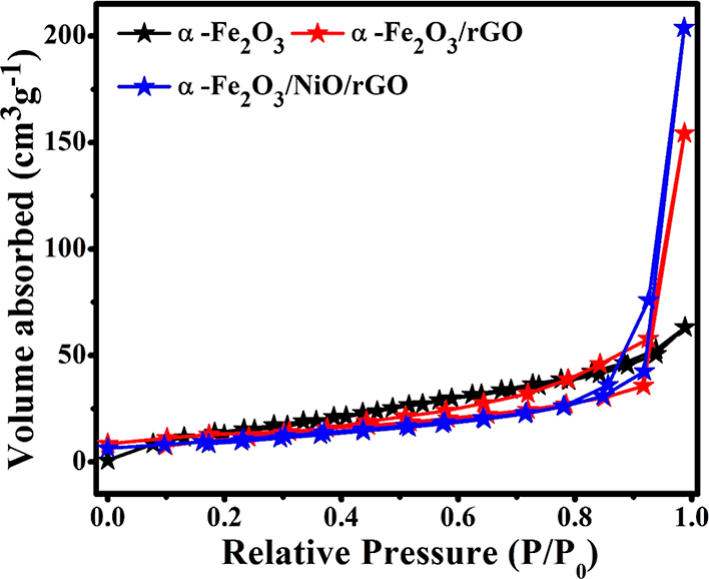
Nitrogen adsorption–desorption isotherms of pure α-Fe_2_O_3_, binary α-Fe_2_O_3_/rGO, and ternary α-Fe_2_O_3_/NiO/rGO composites.

The surface area of the prepared materials was obtained from BET N_2_ adsorption–desorption isotherms in the relative pressure range of 0.0–1.0. Figure 7 shows the nitrogen adsorption–desorption isotherms and BHJ pore size distribution curves of pure α-Fe_2_O_3_, binary α-Fe_2_O_3_/rGO, and ternary α-Fe_2_O_3_/NiO/rGO composites. According to IUPAC classifications, all the prepared materials exhibited that type IV isotherms curves display a distinct hysteresis loop-type H4. The measured surface areas were 80, 132, and 175 m^2^ g^–1^ for pure α-Fe_2_O_3_, binaryα-Fe_2_O_3_/rGO, and ternary α-Fe_2_O_3_/NiO/rGO composites, respectively (Figure 7). In comparison with the surface area of pure α-Fe_2_O_3_ and binary α-Fe_2_O_3_/rGO composites, the surface area of the surface of ternary α-Fe_2_O_3_/NiO/rGO composite increased due to the existence of rGO sheet with smaller NiO nanoparticles in the ternary composite.

### 5.1 Electrochemical Property Analysis

**Figure 8:**
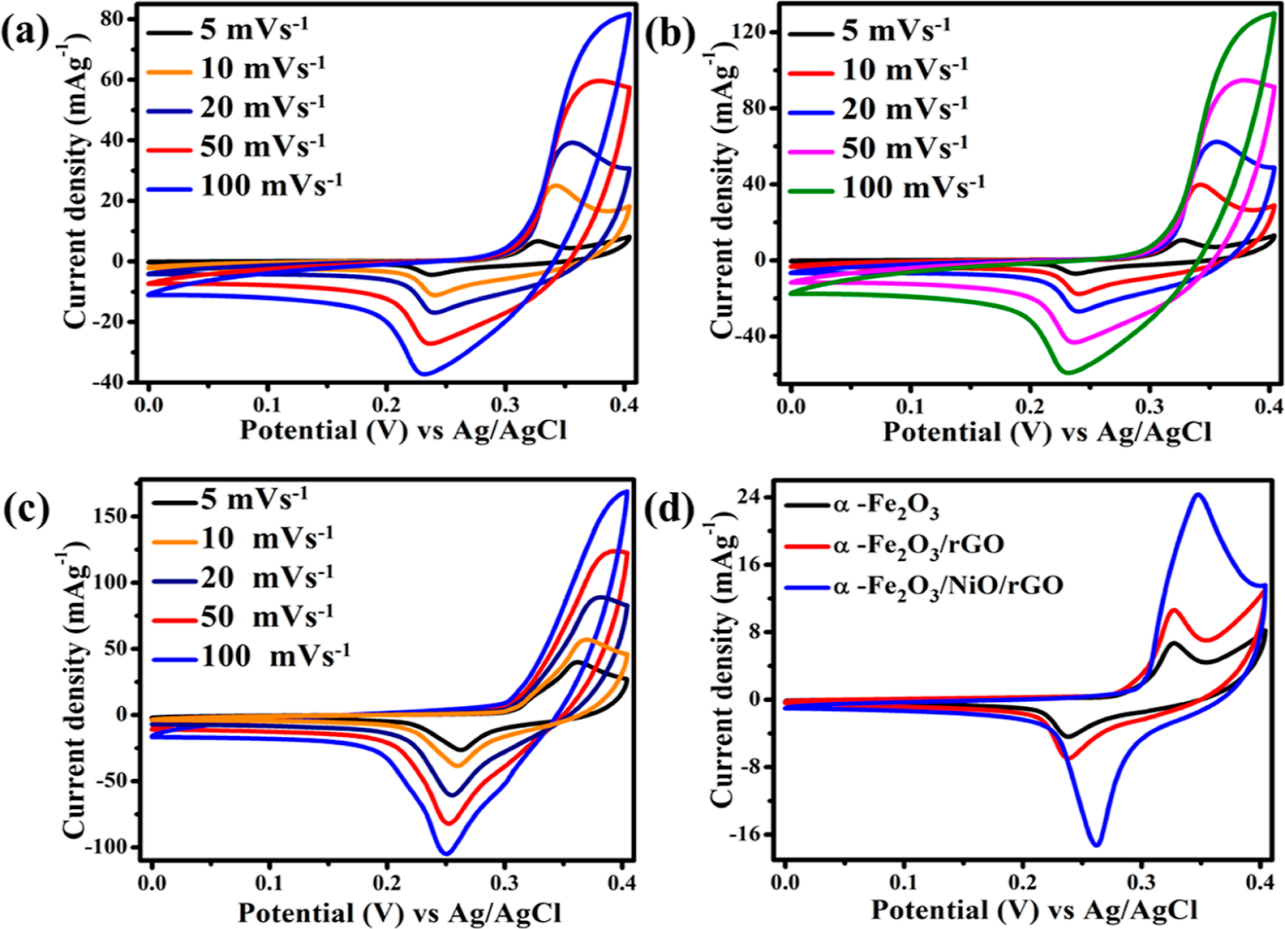
CV curves of (a) pure α-Fe_2_O_3_, (b) binary α-Fe_2_O_3_/rGO, and (c) ternary α-Fe_2_O_3_/NiO/rGO composite at different scan rates between 5 and 100 mV s^–1^ and (d) comparison of CV curves of pure α-Fe_2_O_3_, binary α-Fe_2_O_3_/rGO, and ternaryα-Fe_2_O_3_/NiO/rGO composites at a scan rate of 5 mV s^–1^_._

The electrochemical properties of the electrode materials were examined in a three-electrode electrochemical cell. The CV curves of pure α-Fe_2_O_3_, binary α-Fe_2_O_3_/rGO, and ternary α-Fe_2_O_3_/NiO/rGO composites were recorded in the potential window between 0 and 4 V versus Ag/AgCl for different scanning rates between 5 and 100 mV s^–1^ (Figure 8a–c). All the electrode materials showed the oxidation and reduction peaks, which imply that the electrode materials possess a pseudocapacitance behavior. Moreover, the capacitance characteristics of all samples were governed by a Faradaic redox reaction, that is, the redox peak conversion between different valance states of iron as Fe^3+^/Fe^2+^.^36^ The area under the CV curve and the current density of the redox peaks for ternary α-Fe_2_O_3_/NiO/rGO composites were higher than that of pure α-Fe_2_O_3_ and binary α-Fe_2_O_3_/rGO composite electrode materials. These results were due to the high surface area and improved electrical conductivity, which improved the ion/charge transport properties and increased the electrochemical reaction sites. Also, the redox peaks in the CV curve were broadened for the ternaryα-Fe_2_O_3_/NiO/rGO composite due to the merging of redox peaks related to NiO.^37^ Moreover, the charge storage mechanism in α-Fe_2_O_3_ was explained in KOH solution as Fe_2_O_3_ + 2K^+^ + 2e^–^ ↔ K_2_Fe_2_O_3_.^38^

**Figure 9:**
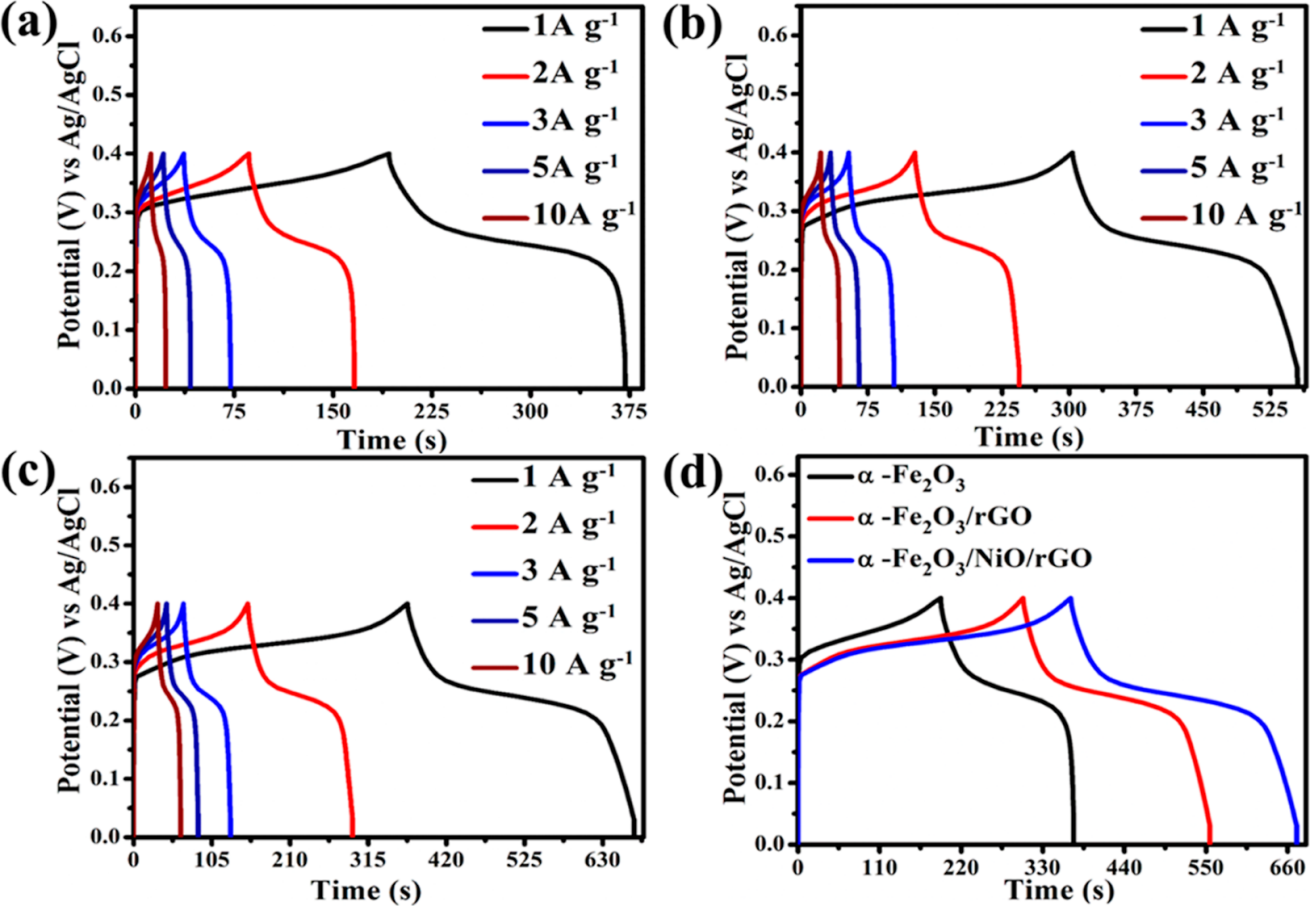
GCD curves of (a) pure α-Fe_2_O_3_, (b) binary α-Fe_2_O_3_/rGO, and (c) ternary α-Fe_2_O_3_/NiO/rGO composites and (d) comparison of charge–discharge curves of α-Fe_2_O_3_ nanoparticles, binary α-Fe_2_O_3_/rGO, and ternary α-Fe_2_O_3_/NiO/rGO composites at a current density of 1 A g^–1^.

**Figure 10:**
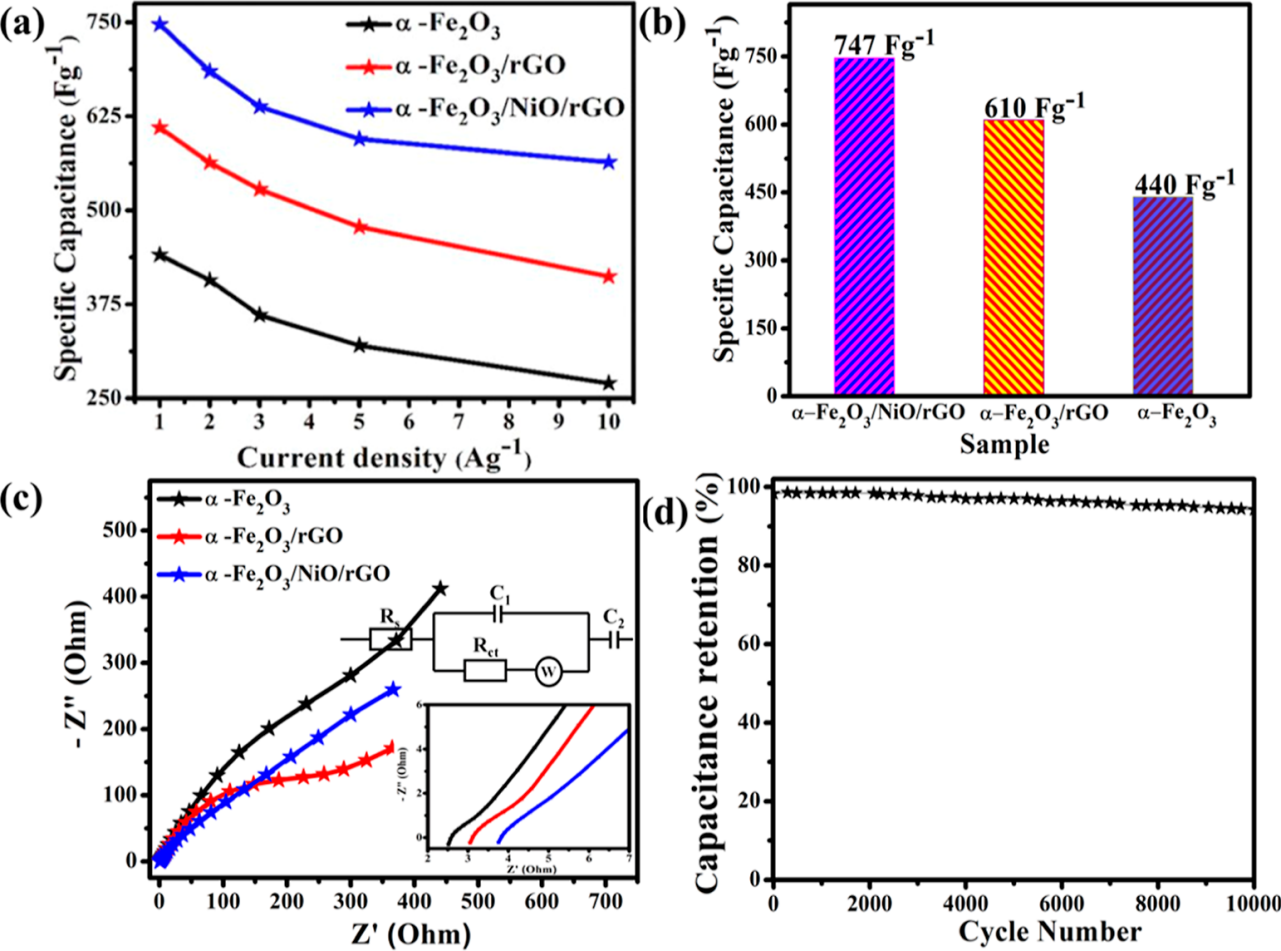
(a) Specific capacitance of pure α-Fe_2_O_3_, binary α-Fe_2_O_3_/rGO, and ternary α-Fe_2_O_3_/NiO/rGO composites at different current densities. (b) Comparison of specific capacitance of pure α-Fe_2_O_3_, binary α-Fe_2_O_3_/rGO, and ternary α-Fe_2_O_3_/NiO/rGO composites at a constant current density of 1 A g^–1^. (c) Nyquist plots of pure α-Fe_2_O_3_, binary α-Fe_2_O_3_/rGO, and ternary α-Fe_2_O_3_/NiO/rGO composites. The inset shows the equivalent circuit model and (d) cycle performance of the ternary α-Fe_2_O_3_/NiO/rGO composite at a current density of 10 A g^–1^.

The GCD measurements were performed for pure α-Fe_2_O_3_, binary α-Fe_2_O_3_/rGO, and ternary α-Fe_2_O_3_/NiO/rGO electrode materials within the potential window of 0–4 V versus Ag/AgCl at different current densities from 1 to 10 A g^–1^ (Figure 9a–c). All the electrode materials exhibited a plateau shape, which is a typical pseudocapacitive behavior, and they are well consistent with the CV curves. Figure 9d illustrates the comparison GCD curves of pure α-Fe_2_O_3_, binary α-Fe_2_O_3_/rGO, and ternary α-Fe_2_O_3_/NiO/rGO electrode materials at a constant current density of 1 A g^–1^. It can be seen that the ternary α-Fe_2_O_3_/NiO/rGO electrode material shows the expanded area and GCD curve, when compared with other electrode materials. As shown in Figure 10a, the specific capacitance of the electrode materials was calculated from the GCD curves of pure α-Fe_2_O_3_, binary α-Fe_2_O_3_/rGO, and ternary α-Fe_2_O_3_/NiO/rGO composite electrode materials for different current densities using eq 1. The highest specific capacitance obtained for the ternary α-Fe_2_O_3_/NiO/rGO electrode material was 747 F g^–1^ at 1 A g^–1^. Even at high current density (10 A g^–1^), the ternary α-Fe_2_O_3_/NiO/rGO composite electrode material showed a highest specific capacitance of 564 F g^–1^ as compared with other electrode materials. The electrochemical reaction kinetics of the prepared electrode materials were studied by EIS measurements at an open-circuit AC potential.

Figure 10c illustrates the Nyquist (*N–q*) plots of pure α-Fe_2_O_3_, binary α-Fe_2_O_3_/rGO, and ternary α-Fe_2_O_3_/NiO/rGO composite electrode materials in the frequency ranging from 100 KHz to 0.01 Hz. The semicircle in the high-frequency region is related to the charge-transfer resistance (*R*_ct_) at the interface of the electrode and the electrolyte, while a straight line in the low-frequency region is Warburg resistance (ZW) caused by the ion diffusion in the electrolyte. Further, the *Z*′ intercept at the *x*-axis is related to the internal resistance (*R*_s_).^36^ The fitting of the *N–q* plots was performed by a modified Randles circuit with a set of resistors and capacitors in series and parallel using a fitting program ZFIT/EC-Lab. The obtained *R*_s_ values were 31.81, 23.43, and 19.2 Ω for pure α-Fe_2_O_3_, binary α-Fe_2_O_3_/rGO, and ternary α-Fe_2_O_3_/NiO/rGO composites, respectively. The ternaryα-Fe_2_O_3_/NiO/rGO composite shows a lowest resistance when compared with other electrode materials. This result could be attributed to high surface area, which provides more reaction sites and excellent conductivity of rGO which increase the electron transport in the composite. The cycling stability is one of the important parameters to find the practical applicability. Figure 10d shows the cyclic stability of ternary α-Fe_2_O_3_/NiO/rGO electrode materials at a current density of 10 A g^–1^. Clearly, the ternary α-Fe_2_O_3_/NiO/rGO electrode material showed a stability of 98% after 10,000 GCD cycles.

**Figure 11:**
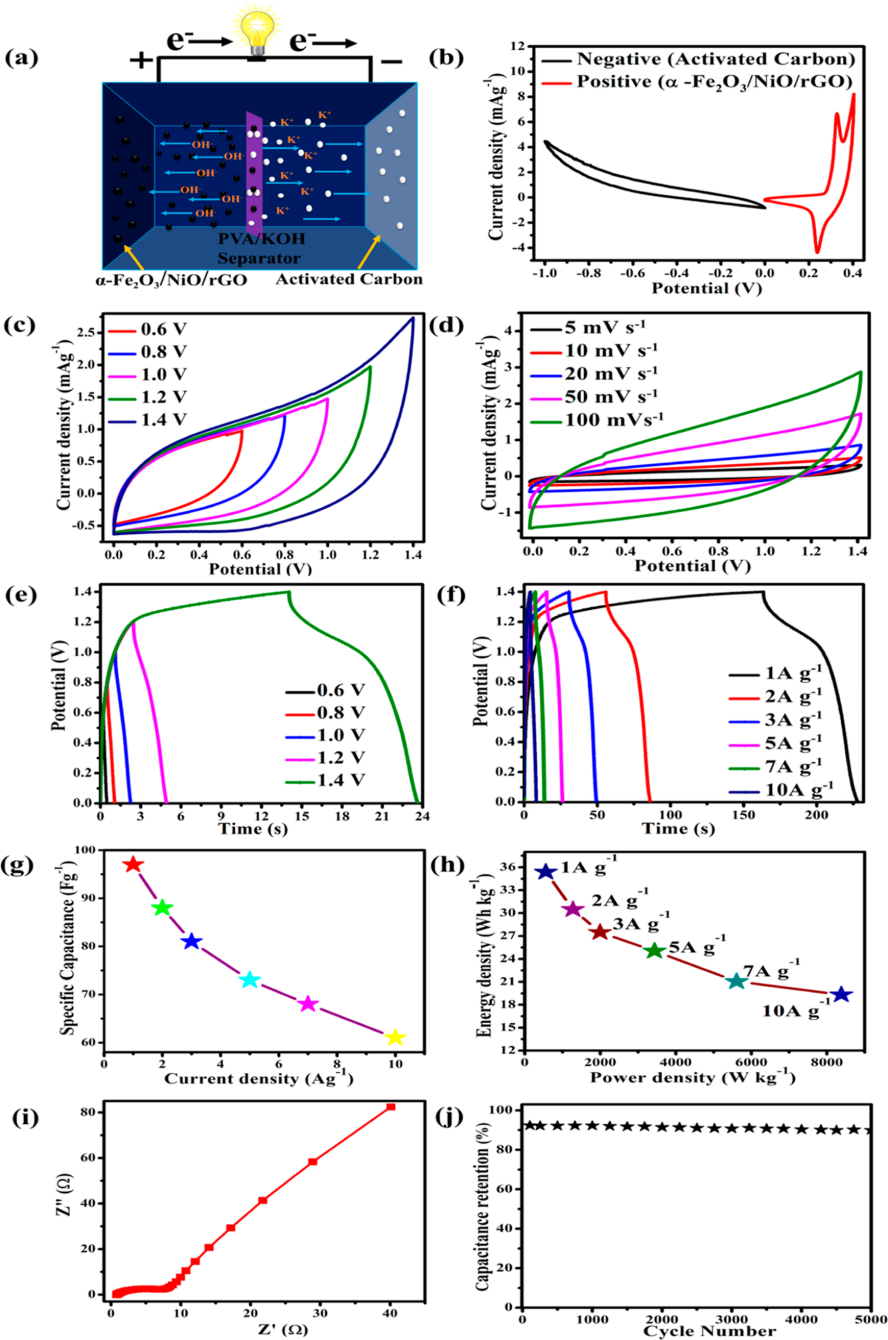
Electrochemical performance of ASC: (a) schematic diagram of the ASCs, (b) CV curves for the positive and negative electrode at a scanning rate of 100 mV s^–1^, (c) CV curves at different potential windows at 100 mV s^–1^, (d) CV curves at different scanning rates between 5 and 100 mV s^–1^, (e) GCD curves for different potential windows at a fixed current density of 7 A g^–1^, (f) comparison of GCD curves at different current densities, and (g) specific capacitance at different current densities. (h) Ragone plots for the as-assembled ASC device, (i) Nyquist plots of a symmetric supercapacitor, and (j) cycle performance of the ASC device at a current density of 10 A g^–1^.

To validate the practical suitability of the ternary α-Fe_2_O_3_/NiO/rGO electrode material, we assembled the ASC device containing a ternary α-Fe_2_O_3_/NiO/rGO electrode as the positive electrode material, AC as the negative electrode material, and KOH-PVA gel as the electrolyte, as shown in Figure 11a. Figure 10b illustrates the CV curves of positive and negative electrodes in a single diagram, which we used to identify the potential window of the ASC device. As shown in Figure 11b, two distinct potential windows can be seen in the range between −1.0 to 0 and 0 to 0.4 V at a scanning rate of 20 mV s^–1^ for negative and positive electrode materials, respectively. This result clearly suggested that the maximum operating potential window of the assembled device is 0.0–1.4 V. Figure 11c shows the CV curves of the ASC device at different potential windows, which provides the operating capability of devices in the potential range of −1.0 to 0.5 V. The CV curves of the ASC device at different scanning rates are shown in Figure 11d, and all the CV curves exhibited a quasi-rectangular shape without any significant changes conforming that the assembled ASC possess a hybrid EDLC and pseudocapacitive behavior. Moreover, the charge storage properties of the ASC device were studied using GCD measurements for different potential windows from 0.6 to 1.4 V (Figure 11e). Moreover, the GCD curves of the assembled ASC device at different current densities from 1 to 10 A g^–1^ were measured and are shown in Figure 11f. On the GCD curves, the charge potential plateau appeared due to the Faradic redox reaction of the working electrode. The specific capacitance (F g^–1^), energy density (*E*, W h kg^–1^), and power density (*P*, W kg^–1^) of the ASC device were calculated using eqs 2–4. Figure 11g represents the calculated specific capacitance of ASC for different current densities. The assembled ASC has the highest specific capacitance of 130 F g^–1^ at a current density of 1 A g^–1^. The calculated current densities and power densities were presented in Figure 11b. The device performance shows a high energy density of 35.38 W h kg^–1^ at a power density of 558.6 W kg^–1^ with an applied current density of 1 A g^–1^. The calculated energy density showed a high value when compared with the already-reported values for α-Fe_2_O_3_-based electrode materials (Table 1). The *N–q* plot of ASC was recorded between 0.01 Hz and 1 kHz at an open-circuit potential, as shown in Figure 11i. The semicircle in the high-frequency region and an oblique line in the low-frequency region indicate that the samples exhibit good capacitive and conductive behavior. Further, the cyclic stability of ASC devices was investigated at a current density of 10 A g^–1^. Approximately 92% of its original capacity was retained even after 5000 GCD cycles.

**Table 1:** Electrochemical Performance of α-Fe_2_O_3_ Electrodes in Non-aqueous Electrolytes

sample name	electrolyte	energy density (W h kg^–1^)	power density (W kg^–1^)	ref. (no.)
core/shell Fe–Ni/Fe_2_O_3_–NiO	NaOH	27.6	6000	(37)
Fe_2_O_3_	Na_2_SO_4_	18	800	(39)
Fe_2_O_3_/GA	KOH	9.8	90.1	(40)
NiO/rGO	KOH	32.5	375	(41)
α-Fe_2_O_3_/NiO/rGO	KOH	35.84	558	this work

## 6 Conclusions

The high-performance ternary α-Fe_2_O_3_/NiO/rGO composite-based electrode material was synthesized by simple chemical methods for supercapacitor application and characterized by an analytical technique such as powder XRD, SEM, TEM, and BET surface area analysis. The ternary α-Fe_2_O_3_/NiO/rGO composite-based electrode material exhibited a high specific capacitance of 747 F g^–1^ at a current density of 1 A g^–1^, when compared with α-Fe_2_O_3_/rGO (610 F g^–1^ @ 1 A g^–1^) and pure α-Fe_2_O_3_ (440 F g^–1^ @ 1 A g^–1^). Moreover, the ternary α-Fe_2_O_3_/NiO/rGO composite electrode material had shown 98% cycling stability of its initial capacitance even after 10,000 charge/discharge cycles. The outstanding electrochemical results of the ternary α-Fe_2_O_3_/NiO/rGO composite electrode material is attributed to the high surface area and improved the electrical conductivity which provided abounded reaction sites and enhanced the electric/ionic transport properties. Further, the ASC device was assembled, and its electrochemical properties were studied. The assembled device showed a high energy density of 35.38 W h kg^–1^ at a power density of 558.6 W kg^–1^ with an applied current density of 1 A g^–1^ and a remarkable cyclic stability up to 5000 cycles.
